# Prevalence of Restless Leg Syndrome and Its Association With Iron Deficiency in Patients With Chronic Kidney Disease: A Cross-Sectional Observational Study

**DOI:** 10.7759/cureus.86188

**Published:** 2025-06-17

**Authors:** Parimala Elangovan, Arulgeetha Murugadhandayuthapany, Dinesh S, Divagar Arivalagan

**Affiliations:** 1 Medicine, Stanley Medical College and Hospital, Chennai, IND; 2 General Internal Medicine, Stanley Medical College and Hospital, Chennai, IND; 3 General Practice, Tagore Medical College and Hospital, Chennai, IND; 4 Medicine, Government Medical College and Hospital, Kallakurichi, Chennai, IND

**Keywords:** chronic kidney disease (ckd), cross sectional studies, iron deficiency anemia (ida), manganese and dopamine transporter, s: restless leg syndrome

## Abstract

Background: Restless leg syndrome (RLS), or Willis-Ekbom disease, is a common yet underdiagnosed neurological disorder characterized by an uncontrollable urge to move the legs, often accompanied by unpleasant sensations. Symptoms are worse during rest and at night, significantly affecting sleep and quality of life. Its prevalence is notably higher among patients with chronic kidney disease (CKD), particularly those undergoing dialysis, and is closely linked with disturbances in iron metabolism. Iron is vital for dopamine synthesis, and its deficiency - common in CKD due to inflammation, poor intake, and blood loss - has been implicated in RLS pathogenesis. This study investigates the prevalence of RLS among CKD patients across various treatment modalities and its association with iron deficiency using serum ferritin, transferrin saturation (TSAT), serum iron, and total iron-binding capacity (TIBC).

Methods: A cross-sectional observational study was conducted at Madras Medical College, Chennai, over six months, involving 150 adult CKD patients (Stages 3-5). Participants were grouped as hemodialysis-dependent (HD), peritoneal dialysis-dependent (PD), conservatively managed (non-dialysis), and post-renal transplant. Patients with non-CKD neurological/psychiatric disorders or those on RLS-inducing medications were excluded. RLS diagnosis was based on the International Restless Legs Syndrome Study Group (IRLSSG) criteria. Clinical interviews, case records, and laboratory tests were used for data collection. Statistical analysis was performed using SPSS (IBM Corp., Armonk, NY, USA), with significance set at p < 0.05.

Results: The average participant age was 51.6 ± 12.3 years; 58% were male. The distribution included HD (40%), conservative (28%), PD (18%), and transplant (14%) groups. RLS was diagnosed in 42% of patients (63 out of 150), with the highest prevalence in HD patients (51.7%), followed by PD (40.7%), conservative (26.2%), and transplant (23.8%). Elderly patients (≥60 years) and females had higher RLS prevalence (46.7% and 47.6%, respectively). Diabetics were more affected than non-diabetics (47.8% vs. 37%).

Patients with RLS had significantly lower iron indices: mean serum ferritin (88.4 ± 25.6 ng/mL vs. 126.7 ± 30.1 ng/mL), TSAT (16.3 ± 4.7% vs. 22.1 ± 5.6%), and serum iron (48.2 ± 11.4 µg/dL vs. 64.7 ± 13.1 µg/dL). TIBC was higher in RLS patients (295 ± 36 µg/dL vs. 273 ± 30 µg/dL). These findings were statistically significant (p < 0.01).

Conclusion: RLS is highly prevalent among CKD patients, especially those on dialysis, and shows a strong association with iron deficiency. Reduced serum ferritin, TSAT, and serum iron levels indicate that impaired iron metabolism contributes significantly to RLS in this population. Functional iron deficiency, even with normal ferritin, may underlie persistent symptoms. Early recognition and targeted iron therapy could reduce RLS burden and improve sleep, mood, and quality of life in CKD patients. Further multicenter studies are needed to validate these findings and develop standardized management protocols.

## Introduction

Restless leg syndrome (RLS), also known as Willis-Ekbom disease, is a neurological sensorimotor disorder characterized by an uncontrollable urge to move legs, typically occurring during rest or inactivity, particularly in the evening or night [1]. It significantly impairs sleep quality and quality of life, often leading to insomnia and psychological distress. Among patients with chronic kidney disease (CKD), particularly those on dialysis, RLS is highly prevalent and frequently underdiagnosed [2]. A 2016 meta-analysis reported a pooled prevalence of 24.2% among CKD patients, with a higher occurrence in those undergoing hemodialysis [3].

The pathophysiology of RLS is multifactorial, with a central role played by iron deficiency. Iron is essential for dopamine synthesis, and a deficit disrupts dopaminergic neurotransmission in the central nervous system, particularly in substantia nigra and basal ganglia [4]. Studies have demonstrated reduced iron concentrations in the brain, particularly in basal ganglia of RLS patients using MRI and CSF analysis, suggesting that brain iron deficiency rather than peripheral levels may be more relevant [5,6]. This dopaminergic dysfunction, triggered by iron loss, leads to the characteristic symptoms of RLS and is further exacerbated in CKD by chronic inflammation, reduced dietary intake, and frequent blood losses during dialysis [7].

Despite its prevalence, RLS is frequently underrecognized in nephrology practice. Diagnostic criteria are largely clinical, and symptoms may be mistaken for uremic neuropathy or peripheral vascular disease. Additionally, few studies have assessed the geographic distribution or modality-specific prevalence of RLS in CKD (e.g., dialysis vs. transplant), creating a gap in the understanding of its epidemiology across patient subgroups [4]. Increasing awareness and routine screening, particularly in those with iron deficiency, may improve detection and guide targeted interventions to alleviate symptoms and enhance quality of life.

## Materials and methods

This cross-sectional observational study was conducted over a period of six months at the Department of General Medicine, Madras Medical College, Chennai, with the primary objective of assessing the prevalence and distribution of RLS among patients with CKD and evaluating its association with iron deficiency across various treatment modalities. A total of 150 patients aged 18 years and above with CKD stages 3 to 5, diagnosed according to Kidney Disease: Improving Global Outcomes (KDIGO) guidelines, were consecutively enrolled from both inpatient and outpatient settings. Participants were retrospectively analyzed and grouped into four categories based on management they had been receiving at the time of data collection: hemodialysis-dependent, peritoneal dialysis-dependent, non-dialysis dependent (optimal medical management with dietary modifications, nutritional supplements, fluid restriction, and regular monitoring), and post-renal transplant recipients. Inclusion criteria included age ≥18 years and informed consent, while patients with neurological disorders unrelated to CKD, psychiatric illness, recent use of RLS-inducing medications, or those who were pregnant or lactating were excluded. Data were obtained through structured interviews, clinical assessments, and laboratory tests. Demographic details, comorbidities, CKD duration, and treatment history were recorded. RLS diagnosis was made using the International Restless Legs Syndrome Study Group (IRLSSG) criteria. Iron parameters including serum ferritin, serum iron, total iron-binding capacity (TIBC), and transferrin saturation (TSAT) were measured, along with routine investigations such as hemoglobin, serum creatinine, and estimated glomerular filtration rate (eGFR). Statistical analysis was performed using SPSS Version 25 (IBM Corp., Armonk, NY, USA). Categorical variables were expressed as frequencies and percentages, and continuous variables as mean ± standard deviation. The Chi-square test was used to compare RLS prevalence across treatment groups, while independent t-tests and ANOVA were employed to assess associations between RLS and iron parameters. A p-value less than 0.05 was considered statistically significant. The study was approved by the Institutional Ethics Committee of Madras Medical College, and informed written consent was obtained from all participants.

## Results

The mean age of the study population was 51.6 ± 12.3 years. There was a male predominance (58%). The distribution of patients across different CKD management modalities was as follows: hemodialysis (40%), peritoneal dialysis (18%), conservative management (28%), and post-transplant (14%). Comorbid conditions such as diabetes mellitus (46%) and hypertension (68%) were prevalent among the participants (Table 1). 

**Table 1:** Demographic and clinical characteristics of the study population. CKD: chronic kidney disease

Parameter	Value
Age (yrs)	51.6 ± 12.3
Sex – Male	87 (58%)
Sex – Female	63 (42%)
Hemodialysis	60 (40%)
Peritoneal Dialysis	27 (18%)
Conservative Management	42 (28%)
Post-Transplant	21 (14%)
Diabetes Mellitus	69 (46%)
Hypertension	102 (68%)
Cardiovascular Disease	33 (22%)
Duration of CKD (yrs)	5.8 ± 3.4
Hemoglobin (g/dL)	9.8 ± 1.2 (normal range 12-16g/dL)

Out of 150 patients, 63 were diagnosed with RLS, giving an overall prevalence of 42%. The prevalence of RLS was higher among females (47.6%) compared to males (37.9%). This gender difference may be attributed to physiological differences in iron metabolism or chronic blood loss during the menstrual cycle. Age-wise distribution showed increasing prevalence with advancing age. The group aged ≥60 years had the highest prevalence at 46.7%, followed by those aged 40-59 years (43.6%), while the lowest prevalence (29.6%) was observed in patients below 40 years. This trend suggests that advancing age may be a risk factor for RLS in CKD. However, this apparent rise in prevalence with increasing age may partly reflect longer CKD duration among older individuals. The highest prevalence of RLS was observed in patients undergoing hemodialysis (51.7%), followed by peritoneal dialysis (40.7%). Patients managed conservatively had a lower prevalence (26.2%), and the lowest prevalence was seen in post-transplant patients (23.8%). The increased prevalence in dialysis patients is likely due to factors such as frequent blood loss, chronic inflammation, and relative iron deficiency. Lower prevalence in transplant and non-dialysis patients may reflect better overall iron balance and metabolic stability. However, since iron indices and inflammatory markers were not compared across groups in our study, these explanations remain speculative.

Among patients with diabetes mellitus, the proportion of RLS was 47.8%, while in non-diabetics it was 37% (Table 2). Diabetes may contribute to RLS through mechanisms such as peripheral neuropathy or vascular insufficiency. 

**Table 2:** Proportion of RLS prevalence across demographic, clinical and treatment-related variables in CKD treatment groups RLS: restless leg syndrome, CKD: chronic kidney disease

Variable	Total (n)	RLS Present (n)	Proportion (%)
Sex			
Male	87	33	37.9%
Female	63	30	47.6%
Age Group			
< 40 years	27	8	29.6%
40–59 years	78	34	43.6%
≥ 60 years	45	21	46.7%
CKD Modality			
Hemodialysis	60	31	51.7%
Peritoneal Dialysis	27	11	40.7%
Conservative Mgmt.	42	11	26.2%
Post-Transplant	21	5	23.8%
Diabetes Mellitus			
Present	69	33	47.8%
Absent	81	30	37.0%

Among the 150 CKD patients, those diagnosed with RLS (n = 63) had significantly lower iron indices compared to those without RLS (n = 87). The mean serum ferritin in the RLS group was 88.4 ± 25.6 ng/mL, while the non-RLS group had a mean ferritin of 126.7 ± 30.1 ng/mL. Similarly, the mean TSAT was 16.3 ± 4.7% in the RLS group, compared to 22.1 ± 5.6% in the non-RLS group. Serum iron levels were also reduced in the RLS group (48.2 ± 11.4 µg/dL) relative to the non-RLS group (64.7 ± 13.1 µg/dL). TIBC was mildly elevated in the RLS group (295 ± 36 µg/dL) compared to the non-RLS group (273 ± 30 µg/dL), reflecting a more pronounced functional iron deficiency. These results support the hypothesis that iron deficiency plays a central role in the pathophysiology of RLS in CKD patients. Notably, a TSAT of <20% and ferritin <100 ng/dL are commonly used thresholds to define iron deficiency in the CKD population. A significant proportion of RLS patients in our study fell below these thresholds. The reduced availability of iron, especially in forms accessible to the central nervous system, may impair dopaminergic neurotransmission and contribute to the sensory-motor symptoms of RLS. The association between low TSAT and serum ferritin with RLS was statistically significant (p < 0.01), consistent with existing literature [4,5] emphasizing brain iron deficiency as a core contributor to RLS. This finding reinforces the importance of routine iron profiling and early correction of iron deficiency in managing CKD-associated RLS (Table 3).

**Table 3:** Comparison of iron profile parameters between RLS and non-RLS groups in CKD patients. RLS: restless leg syndrome, CKD: chronic kidney disease, TSAT: transferrin saturation, TIBC: total iron-binding capacity

Parameter	RLS Group (n = 63)	Non-RLS Group (n = 87)	P-Value
Serum Ferritin (ng/mL)	88.4 ± 25.6	126.7 ± 30.1	< 0.01
Transferrin Saturation (TSAT) (%)	16.3 ± 4.7	22.1 ± 5.6	< 0.01
Serum Iron (µg/dL)	48.2 ± 11.4	64.7 ± 13.1	< 0.01
Total Iron-Binding Capacity (TIBC) (µg/dL)	295 ± 36	273 ± 30	< 0.01

## Discussion

In our study, we found that CKD patients with RLS exhibited significantly lower iron indices-including serum ferritin, TSAT, and serum iron-compared to those without RLS. These findings are consistent with existing literature highlighting iron deficiency as a key factor in the pathophysiology of RLS [8].

The mean serum ferritin level in our RLS group (88.4 ± 25.6 ng/mL) was significantly lower than that in the non-RLS group (126.7 ± 30.1 ng/mL). This is in agreement with the meta-analysis by Li et al., which encompassed 907 RLS patients (220 with augmentation and 687 without) and confirmed that low ferritin levels are associated with an increased risk of RLS in various populations, including those with chronic illnesses like CKD [8]. However, some studies have reported no significant difference in ferritin levels between RLS and non-RLS patients, suggesting that serum ferritin alone may not be a reliable indicator in the presence of systemic inflammation [8].

The pathophysiological mechanism linking iron deficiency and RLS involves impaired dopaminergic neurotransmission in the central nervous system. Iron serves as a cofactor for tyrosine hydroxylase, the rate-limiting enzyme in dopamine synthesis. Iron deficiency leads to downregulation of this enzyme, reduced dopamine production, and alterations in dopamine receptor sensitivity, particularly in the substantia nigra and putamen, regions implicated in sensorimotor integration [9,10]. Furthermore, iron deficiency in the human brain has been shown to increase glutamatergic activity, contributing to hyperexcitability in sensorimotor pathways [11]. In CKD, systemic inflammation upregulates hepcidin, which inhibits ferroportin-mediated iron export from enterocytes and macrophages, reducing iron bioavailability even in the presence of adequate stores. This results in functional iron deficiency, which has been implicated in the pathogenesis of RLS [12]. MRI studies in RLS patients have demonstrated reduced brain iron concentrations, particularly in the thalamus and substantia nigra, supporting the central role of brain iron deficiency in RLS pathophysiology [5]. These findings may inform future diagnostic strategies by promoting the use of advanced neuroimaging to detect central iron deficits, and could also guide the development of targeted treatments aimed at restoring iron levels specifically within the central nervous system.

Our finding of reduced TSAT (16.3 ± 4.7% vs. 22.1 ± 5.6%) aligns with the observations by Riar et al., who emphasized TSAT as a more sensitive indicator of iron availability for neurological function in RLS patients, particularly in CKD, where ferritin can be elevated due to inflammation [13]. In contrast, a study by Riar et al. in pediatric CKD patients did not find a consistent association between TSAT and RLS symptoms, indicating potential age-related or disease-stage-related differences [10].

The lower serum iron levels (48.2 ± 11.4 µg/dL) and higher total iron-binding capacity (295 ± 36 µg/dL) in our RLS group suggest a functional iron deficiency pattern. This observation corroborates findings from Allen et al., who demonstrated disrupted iron transport and storage in RLS patients despite normal ferritin levels [11]. The elevated TIBC observed in our RLS group may reflect an adaptive response to iron deficiency, aiming to increase iron binding and transport, as previously suggested in studies on iron metabolism in uremic patients [13].

Furthermore, chronic inflammation in CKD is known to raise hepcidin levels, which impairs intestinal iron absorption and traps iron in storage sites, contributing to functional iron deficiency. This mechanism may explain why RLS symptoms persist despite apparently adequate ferritin levels, as described by Rizzo et al., who found elevated hepcidin in RLS patients [5]. Our results support this concept, reinforcing the importance of interpreting ferritin in the context of TSAT and TIBC.

Collectively, our findings highlight that low TSAT and serum ferritin are significantly associated with the presence of RLS in CKD patients, underscoring the role of iron deficiency in its pathophysiology. These results emphasize the clinical value of routine iron profiling and early correction of iron deficiency in CKD patients to reduce RLS symptom burden.

Despite the valuable insights provided, this study has several limitations. Firstly, its cross-sectional design limits the ability to establish causality between iron deficiency and the development of RLS. Secondly, the sample size, though adequate for preliminary observations, was relatively small and drawn from a single tertiary care center, potentially limiting the generalizability of the findings to broader CKD populations, including those in rural or primary care settings. Thirdly, we did not measure serum hepcidin levels, which could have provided a more comprehensive understanding of iron regulation and its role in functional iron deficiency in CKD. Additionally, inflammatory markers such as C-reactive protein (CRP) were not assessed, which might have helped in better interpreting ferritin levels in the context of systemic inflammation. Therefore future studies should consider assessing CRP to help differentiate between true and functional iron deficiency due to inflammation. Additionally, potential confounding factors such as age, sex, CKD duration, comorbidities, and medication use were not adjusted for in our analysis. The interpretations of the whole study were based only on peripheral iron levels, and investigations were not taken to assess brain iron levels. Furthermore, RLS diagnosis was based on self-reported symptoms using the IRLSSG criteria, which may be subject to recall bias and subjective interpretation. Finally, the study did not evaluate the impact of iron supplementation or dopaminergic therapy on RLS symptoms, which could have further clarified the clinical relevance of the observed associations.

## Conclusions

In conclusion, our study highlights a significant association between iron deficiency and the presence of RLS in patients with CKD. CKD patients diagnosed with RLS demonstrated notably lower serum ferritin, transferrin saturation, and serum iron levels, along with higher TIBC values, indicating both absolute and functional iron deficiency. These findings support the growing body of evidence suggesting that disrupted iron homeostasis plays a pivotal role in the pathogenesis of RLS.

Given the burden of RLS on sleep quality, mental health, and overall quality of life in CKD patients, early identification and correction of iron deficiency should be prioritized. Routine monitoring of iron indices, including ferritin, TSAT, and TIBC, may aid in the timely diagnosis and effective management of RLS in this vulnerable population. Further longitudinal and interventional studies are warranted to evaluate whether targeted iron therapy can mitigate RLS symptoms and improve patient outcomes in CKD. A potential future approach could also involve a randomized controlled trial comparing intravenous iron supplementation versus placebo in CKD patients with RLS and confirmed iron deficiency.

## References

[1] Allen RP, Picchietti DL, Garcia-Borreguero D (2014). Restless legs syndrome/Willis-Ekbom disease diagnostic criteria: updated International Restless Legs Syndrome Study Group (IRLSSG) consensus criteria--history, rationale, description, and significance. Sleep Med.

[2] Safarpour Y, Vaziri ND, Jabbari B (2023). Restless legs syndrome in chronic kidney disease- a systematic review. Tremor Other Hyperkinet Mov (N Y).

[3] Lin Z, Zhao C, Luo Q, Xia X, Yu X, Huang F (2016). Prevalence of restless legs syndrome in chronic kidney disease: a systematic review and meta-analysis of observational studies. Ren Fail.

[4] Earley CJ, Connor JR, Beard JL, Malecki EA, Epstein DK, Allen RP (2000). Abnormalities in CSF concentrations of ferritin and transferrin in restless legs syndrome. Neurology.

[5] Rizzo G, Li X, Galantucci S, Filippi M, Cho YW (2017). Brain imaging and networks in restless legs syndrome. Sleep Med.

[6] (2015). MRI measurement of brain iron in patients with restless legs syndrome. Neurology.

[7] Higuchi T, Abe M, Mizuno M (2015). Association of restless legs syndrome with oxidative stress and inflammation in patients undergoing hemodialysis. Sleep Med.

[8] Li YS, Yeh WC, Hsu CY (2023). Association of low serum ferritin levels with augmentation in patients with restless legs syndrome: a systematic review and meta-analysis. Sleep Med.

[9] Manconi M, Garcia-Borreguero D, Schormair B, Videnovic A, Berger K, Ferri R, Dauvilliers Y (2021). Restless legs syndrome. Nat Rev Dis Primers.

[10] Riar SK, Leu RM, Turner-Green TC (2013). Restless legs syndrome in children with chronic kidney disease. Pediatr Nephrol.

[11] Allen RP, Barker PB, Horská A, Earley CJ (2013). Thalamic glutamate/glutamine in restless legs syndrome: increased and related to disturbed sleep. Neurology.

[12] Ganz T (2013). Systemic iron homeostasis. Physiol Rev.

[13] Riar SK, Greenbaum LA, Bliwise DL, Leu RM (2019). Restless legs syndrome in chronic kidney disease: is iron or inflammatory status to blame?. J Clin Sleep Med.

